# Translation and psychometric testing of the sense of competence in Dementia Care Staff Scale in Chinese amongst dementia care staff in nursing homes of China

**DOI:** 10.1186/s12877-023-03845-x

**Published:** 2023-03-21

**Authors:** Xueli Bian, Jing Wang, Junqiao Wang

**Affiliations:** 1 School of Nursing, Fudan University, Shanghai, China; 2 Department of Nursing, Zhongshan Hospital, Fudan University, Shanghai, China

**Keywords:** Cognitive impairment, Dementia, Competence, Nursing care aides, Caregiver, Chinese version scale, Reliability, Validity

## Abstract

**Background:**

Although China has the largest population of persons with dementia, there is no validated tool available to accurately assess formal caregivers’ competence in dementia care in long-term care settings. Appropriately assessing nursing staff’s level of competence in dementia care is the first step to develop precision training interventions to improve the quality of dementia care. The Sense of Competence in Dementia Care Staff scale (SCIDS) is a user-friendly tool with satisfactory reliability and validity. We adapted SCIDS into a Chinese version (SCIDS-C) and validated its uses in China’s socio-cultural context to assess nursing staff’s capability and competence in dementia care at nursing homes.

**Aims:**

We aimed to adapt and psychometrically test the tool among frontline nursing staff in long-term care settings in China.

**Methods:**

The research employed a correlational design with repeated measures. In translation section, we adapted and tailored the original scale in the cultural and social context in China’s nursing homes. The scale’s adaptation consists of translating adaptation and semantic equivalence. In psychometric testing phase, we tested the validity and reliability of the scale with 174 nursing staff conveniently from six nursing homes. Construct validity was tested using exploratory factor analysis (EFA), including principal component analysis and maximum variance rotation method. Reliability was tested using Cronbach’s alpha value and intraclass correlation coefficient (ICC).

**Results:**

The SCIDS-C has 17 items, which belong to the two sub-scales, the Relationship-Centered Care(RCC) and Professional Care(PC). The Cronbach’s alpha value was 0.88, showing a good internal consistency. The full scale’s value of ICC was 0.94 which indicated good reliability. Exploratory factor analysis(EFA) extracted 2 common factors in each sub-scale, cumulative variance contribution rate was 56.71% and 53.92%, respectively. The named four factors are the same as the Sense of Competence in Dementia Care Staff (SCIDS) scale in English, including Building Relationships, Sustaining Personhood, Professionalism and Care Challenges.

**Conclusion:**

The SCIDS-C has shown good reliability and validity. It can be used as an appropriate tool to evaluate the competence of nursing care staff to provide dementia care for residents in nursing homes.

## Background

Dementia is an advanced cognitive impairment syndrome. It can lead to the reduction of patient's ability of daily living, social communication and can be accompanied by mental and behavioral symptoms, which is the main cause of disability and dependence [1]. Dementia is not simply a neurological disorder, but also a long-term care and social adaptation issue in public health [2]. In China, the number of the patients with dementia (PWD) reached 15.07 million in 2018, accounting for a quarter of the population with dementia around the world [3]. Dementia is a progressive condition yet its progression tends to be personalized in individuals and almost all patients will experience psycho-behavioral symptoms in different ways [4, 5]. In addition to the decline in memory, language expression, visual space perception, and executive ability caused by cognitive decline, as the disease progresses, the clinical syndrome of PWD can show various behavioral and psychological symptom of dementia (BPSD), including apathy, depression, restlessness, anxiety, personality changes, and mental symptoms as well as abnormal behaviors such as wandering, aggressive behavior, sleep disorder, inappropriate sexual behaviors, and refusal to care (Oliveira et al., [6]). Residents with BPSD tend to have lower quality of life, progressive dysfunction, and increased mortality rate (Kales et al., [7]).

BPSD not only have a significant impact on the health and quality of life of patients, but they also cause a huge physical and mental burden on caregivers. Compared with non-dementia patients, nurses have heavier and greater responsibility for caring for patients with dementia. Whether it is a formal caregiver or an informal caregiver, caring for PWD is a long-term and difficult nursing task, especially if the nurses are not equipped with the care knowledge about BPSD or without much relevant work experience [8]. The condition of dementia is relatively complex and latent, which means that early symptoms are difficult to identify. If the correct nursing intervention is not received, the patient’s cognitive deterioration will accelerate and it is difficult to reverse. Persons with advanced dementia are usually institutionalized [8]. Dementia caregivers in long-term care institutions need to have higher nursing skills to deal with the complicated symptoms in PWD. Moreover, handling BPSD appropriately can delay the deterioration of the cognitive ability of PWD and reduce the occurrence of complications, but most of the nursing staff in Chinese long-term care institutions are laid-off workers, rural women and even the elderly with limited education [9]. The care staff without care knowledge or work experience have many misunderstandings about the mental and behavioral symptoms of dementia residents, which discourage their sense of competence in dementia care [10].

Nursing teams experience a high level of stress and caregiving burden when providing care for older residents with dementia. Competence of nursing staff in dementia care is not only essential to improve quality of care for older residents with dementia in nursing homes, but also to improve quality of their own work and life [11]. Competence in an area of clinical practice is understood to include knowledge, skills, and attitudes (KSAs) of dementia care [12]. Among nursing staff, if the caregivers lack correct BPSD nursing skills that could respond negatively to the caregiving needs, it will cause serious outcome such as depression and mental disorders and affect their life and work [11]. Lack of competence amongst staff in dementia care may contribute to increased staff turnover and decreased the quality of care. Appropriately assessing nursing staff’s competence in dementia care can provide substantial implications for developing appropriate training programs and evaluate the effectiveness of the programs.

However, most available tools measuring care competence targeted family caregivers. Tools that targeted formal dementia caregivers mainly focused on their dementia care knowledge, care attitude and care work burden separately [13–16]. Few research explored the evaluation of competence in dementia care among frontline nursing staff. There is no validated tool available to accurately assess staff’s competence in dementia care in the long-term care settings in China. Schepers et al. developed a scale dedicated to assessing the Sense of Competence in Dementia Care Staff (SCIDS), including building relationships, sustaining personhood, professionalism, and care challenges. At present, the scale has been used to evaluate the effectiveness of training programs and has shown its practicability and applicability [17–19]. Thus, we decided to adapt SCIDS into a Chinese version and validate it in China’s social and cultural context to assess staff’s competence in dementia care in long-term care setting.

## Aims

The aim of the study was to adapt the scale into Chinese context and test the psychometric properties of the SCIDS scale amongst frontline care staff in nursing homes. We used the guidelines for the process of cross-cultural adaptation of self-report measures and the report of the ISPOR task force and the Guideline for Reporting Reliability and Agreement Studies (GRRAS) in the research [20–22].

## Methods

### SCIDS scale

The original SCIDS was compiled by Schepers et al. in 2012 with 17 items, using the Likert four-level scoring method and measuring each item by “not at all”, “little bit”, “quite a lot”, “very much”. The scale consists of 4 parts, including building relationships(Item 1, 2, 3, 4), sustaining personhood(Item 5, 6, 11, 16), and professionalism(Item 7, 8, 9, 10, 12), care challenges(Item 13, 14, 15, 17). Items can be divided into two sub-scales, which are Relationship-Centered Care(RCC) and Professional Care(PC). The former includes building relationships and sustaining personhood; the latter includes professionalism and care challenges. The scale has a total score of 17 to 68 points. The higher the score, the higher the nurses’ competence in dementia care work. The Cronbach’s alpha value of the SCIDS scale in English was 0.91, indicating that the scale has good reliability.

## Phase 1: Translation and adaption of the SCIDS scale

The scale was analyzed according to the guidelines for the process of cross-cultural adaptation of self-report measures and the report of the ISPOR task force for translation and cultural adaptation [20, 22]. It was conducted between September 2020 and October 2020. Firstly, we obtained approval from the developer of the scale via email. The SCIDS scale was forward-translated from English into Chinese (T1, T2) by two bilingual translators, called key in-country persons, who had experience living in China and Australia. One translator was a professor specialized in nursing for the aged and nursing educating with over 10-year experience working in China. Another was a registered nurse with over 5-year experience working in both China and Australia and was an expert in dementia care of nursing homes. The research team, consisting of two geriatric care professionals, compared the two translated SCIDS-C versions and examined discrepancies between terms, sentences, and meanings. Researchers replaced relevant sentences without changing the original expressions or means until the consensus was reached.

The post-forward SCIDS-C scale translation (T12) was blind translated back into English by an independent translator with experience of living in China and USA, who had no previous exposure to the original English SCIDS scale. The research team members compared the backward translation version to the original English SCIDS scale and revised discrepancies. Meanwhile, researchers evaluated whether the back translation correctly reflected the intended meanings of the original until backward translation of the SCIDS scale was acceptable to the research team members. Linguistic validation evaluated how dementia care staff understand and respond to the adapted scale so that to arrive semantic equivalence through the pilot survey and cognitive interviewing in dementia care staff of nursing homes.

## Phase 2: Psychometric testing of the SCIDS-C scale

It was conducted between October 2020 and December 2020. Construct validity was tested by the internal structure of the scale, using the exploratory factor analysis(EFA) which includes the principal component analysis and maximum variance rotation method to extract the factors comparing to the English scale. The Guideline for Reporting Reliability and Agreement Studies (GRRAS) was used to test the reliability of the SCIDS-C scale by psychometric methods [21]. The reliability was assessed by Cronbach’s alpha value and intraclass correlation coefficient (ICC). The test–retest reliability was checked by re-administration to the same people at an interval of one-week [23–27]. SPSS 25.0 software was used for statistical analysis of the data.

## Data collection

Informed consent from each participant was obtained prior to data collection. After signed consent form was received, data collection form was distributed to potential participant. Upon completion, the questionnaires were returned to the researchers with the date on it. The data collection form was a questionnaire including two parts: demographic data and the SCIDS-C scale.

### Ethical considerations

The research’s ethical approval was obtained from ethics board of School of Nursing, Fudan University (IRB#2020–05-06). Approval for the translation of the SCIDS scale and access permission were obtained by the scale developer.

## Results

### Demographic characteristic

The SCIDS-C scale is the care competence measurement of the frontline dementia care staff in nursing homes. Thus, this study used convenience sampling, involved 174 nursing staff in six nursing facilities of Shanghai. The questionnaires were not recorded with names to ensure confidentiality. Demographics of the participants are present in Table 1 (See Table 1).

**Table 1:** Demographics of participants

Demographic and characteristic	N(%)
**Institution**	
Public nursing facility	78(44.83)
Public–private nursing facility	75(43.10)
Private nursing facility	21(12.07)
**Gender**	
Male	19(10.92)
Female	155(89.08)
**Age group**	
30 ~ 39 years old	4(2.30)
40 ~ 49 years old	57(32.76)
50 ~ 59 years old	104(59.77)
60 years old and over	9(5.17)
**Education**	
Primary school	65(37.36)
Middle school	74(42.53)
High school/Technical school	35(20.11)
**Marital status**	
Spousal	163(93.68)
Single	11(6.32)
**Work experience**	
Under 1 year	35(20.11)
1 ~ 5 years	74(42.53)
6 ~ 10 years	40(22.99)
11 ~ 15 years	25(14.37)
**Training experience**	
Yes	107(61.49)
No	67(38.51)
**Monthly salary**	
Under 3000	27(15.51)
3000 ~ 5000	135(77.59)
Over 5000	12(6.90)
**Certification**	
Primary	142(81.61)
Middle and over	32(18.39)

## Results of translation and adaptation

The SCIDS scale was translated from English into Chinese. Pilot research was conducted with 10 respondents who had more than 1 year working experience in nursing homes. After completing the scale, respondents were interviewed regarding the expression of each item, whether they could get the correct meaning of each item. Then semantic equivalence of the two versions was examined. In the translation process, a few items were revised to reduce ambiguity. For example, the forward translation of item 3, “how well do you feel you can engage a person with dementia in a conversation”, was first translated as “how well do you feel you can encourage people with dementia to participate in the conversation” in Chinese. However, in English, the word “engage” refers to succeeding in attracting and keeping someone’s attention and interest. In forward translation, the phrase “encourage people with dementia to participate in the conversation” means that caregivers try to attract the people with dementia to join in the conversation, but we are unsure whether they decide to join or stay in the conversations. Based on the discussions amongst researchers, item 3 was translated into “how well do you feel you can let people with dementia actively participate in the conversation”, which means caregivers could be able to attract the people with dementia into conversation successfully. Meanwhile, item 12, “how well do you feel you can deal with personal care, such as incontinence in a person with dementia”, was blind back-translated into “how well do you feel you can provide daily care, such as helping patients with dementia clean up their excretions”. Based on the research team’s discussion, “incontinence” was kept because “excretion” is different from “incontinence” in definition.

## Results of psychometric testing

Construct validity was tested by EFA. The items were divided into Relationship-Centered Care(RCC) and Professional Care(PC). KMO and Bartlett test was calculated by SPSS 25.0, which KMO > 0.50 and Bartlett test value < 0.05 means the good construct validity of the version of Chinese scale [21]. KMO and Bartlett test confirmed that it is possible to apply this scale (KMO = 0.79, 0.83, respectively; p value < 0.001. ). Principal component analysis and maximum variance rotation method in RCC defined two factors(considering the method of the eigenvalues > 1) with 56.71% explained variance. One of the factors in RCC is building relationships, including items 1, 2, 3, 4; another factor is sustaining personhood, including item 5, 6, 11, 16. Principal component analysis and maximum variance rotation method in PC defined two factors(considering the method of the eigenvalues > 1) with 53.92% explained variance. One of the factors in PC is professionalism, including item 7, 8, 9, 10, 12; another factor is care challenges, including item 13, 14, 15, 17. Every factor loading analysis in sub-scale was recorded in Table 2 (See Table 2).

**Table 2:** Exploratory factor analysis of SCIDS-C

**Item**	RCC sub-scale (*N* = 174)		PC sub-scale (*N* = 174)	
**How well do you feel you can**	Building relationships	sustaining personhood	professionalism	care challenges
1. understand the feelings of a person with dementia?	0.85			
2. understand the way a person with dementia interacts with the people and things around them?	0.80			
3. engage a person with dementia in a conversation?	0.86			
4. balance the needs of the person with dementia with their relative’s wishes and the service’s limitations?	0.53			
5. use information about their past (such as what they used to do and their interests), when talking to a person with dementia?		0.72		
6. change your work to match the changing needs of a person with dementia?		0.70		
11. protect the dignity of a person with dementia in your work?		0.65		
16. offer choice to a person with dementia in everyday care (such as what to wear, or what to do)?		0.52		
7. keep up a positive attitude towards the people you care for?			0.61	
8. keep up a positive attitude towards the relatives of a person with dementia?			0.55	
9. keep yourself motivated during a working day?			0.79	
10. play an active role in your staff team?			0.81	
12. deal with personal care, such as incontinence in a person with dementia?			0.54	
13. deal with behavior that challenges in a person with dementia?				0.66
14. decide what to do about risk (such as harm to self or others) in a person with dementia?				0.71
15. offer stimulation (for the mind, the senses and the body) to a person with dementia in your daily work?				0.75
17. engage a person with dementia in creative activities during your normal working day?				0.71

Cronbach’s alpha value was calculated to evaluate the internal consistency in each of the two sub-scales. The Cronbach’s alpha value > 0.70 indicate a satisfactory result [26]. Table 3 shows Cronbach’s alpha values in case the item in question was excluded. In both scales, deleting each item decreased the entire Cronbach alpha value. Next, the Cronbach’s alpha value was calculated for each of the sub-scale as well as for the entire scale. The entire scale’s Cronbach alpha value was 0.88, showing a good internal consistency. 25 participants from different nursing homes were invited to help us evaluate test–retest reliability with a one-week interval. The ICC was tested for the full scale (ICC = 0.94,*p <* 0.000,SEM = 0.28), RCC(ICC = 0.92,*p <* 0.000,SEM = 0.93), PC(ICC = 0.89,*p <* 0.000,SEM = 0.06), building relationships(ICC = 0.88,*p <* 0.000,SEM = 3.16),sustaining personhood(ICC = 0.86,*p <* 0.000,SEM = 0.24), professionalism(ICC = 0.83,*p <* 0.000, SEM = 0.06) and care challenges (ICC = 0.91,*p <* 0.000, SEM = 0.15) (See Table 4).

**Table 3:** Item analysis (17 items)

**Item** **How well do you feel you can**	Cronbach’s alpha if item deleted
**RCC sub-scale**	
**Building relationships**	
1. understand the feelings of a person with dementia?	0.87
2. understand the way a person with dementia interacts with the people and things around them?	0.87
3. engage a person with dementia in a conversation?	0.87
4. balance the needs of the person with dementia with their relative’s wishes and the service’s limitations?	0.71
**Sustaining personhood**	
5. use information about their past (such as what they used to do and their interests), when talking to a person with dementia?	0.87
6. change your work to match the changing needs of a person with dementia?	0.87
11. protect the dignity of a person with dementia in your work?	0.87
16. offer choice to a person with dementia in everyday care (such as what to wear, or what to do)?	0.87
**PC sub-scale**	
**Professionalism**	
7. keep up a positive attitude towards the people you care for?	0.87
8. keep up a positive attitude towards the relatives of a person with dementia?	0.87
9. keep yourself motivated during a working day?	0.87
10. play an active role in your staff team?	0.87
12. deal with personal care, such as incontinence in a person with dementia?	0.87
**Care challenges**	
13. deal with behavior that challenges in a person with dementia?	0.87
14. decide what to do about risk (such as harm to self or others) in a person with dementia?	0.86
15. offer stimulation (for the mind, the senses and the body) to a person with dementia in your daily work?	0.86
17. engage a person with dementia in creative activities during your normal working day?	0.87

**Table 4:** Reliability analysis of the SCIDS-C scale (17 items)

	**Items**	**Cronbach’s α**	**ICC**
**SCIDS-C scale**	17	0.88	0.94
**RCC sub-scale**	8	0.80	0.92
Building relationships	4	0.82	0.88
Sustaining personhood	4	0.63	0.86
**PC sub-scale**	9	0.81	0.89
Professionalism	5	0.75	0.83
Care challenges	4	0.72	0.91

## Discussion

Analysis of the SCIDS-C scale revealed a satisfactory reliability and validity. The Cronbach’s alpha value of the SCIDS-C scale was 0.88, which is comparable with the developer’s result (0.91). The ICC of the SCIDS-C ranged from 0.83 to 0.94(*P <* 0.001), indicating the good reliability. However, one-week interval seems relatively short. During the research, we interviewed the respondents before first test and retest, there was no difference in the familiarity of the question between the two sides of the test. Upon examination of these items, deletion did not increase the reliability coefficients (Cronbach’s alpha value) of the SCIDS-C. Hence, the items were all retained because their assessments of some areas were comparable with that of the original SCIDS scale. The SCIDS-C’s internal consistency was satisfactory, and the items measured the intended features consistently.

Almost all the participants mentioned the importance of building relationships with residents to understand the personhood and individual characteristic of the PWD. Furthermore, most of the dementia care staff lacked the competence in managing behavioral and psychological symptom of dementia. RCC sub-scale and PC sub-scale were formed with the development of dementia care practice. As well as the original scale, principal component analysis and maximum variance rotation method divided the 17 items by four factors, including building relationships, sustaining personhood, professionalism, and care challenge.

Relationship-Centered Care (RCC) is a core component of the tool. RCC is a critical part of person-centered dementia care. It can be originated from the work of Carl Rogers, which focused on individual personal experience as the basis and standard for living and therapeutic effect [28]. Tom Kitwood firstly used the term in dementia care to bring together ideas and ways of working that emphasized communication and relationships [29]. Nursing staff are central in developing meaningful relationships with PWD. RCC required nursing staff to not only have knowledge of dementia care, but also obtain communicative skills to build reciprocal relationships with PWD. However, task-orientated approach is still commonly used in long-term care settings. PWD are often left feeling isolated, disempowered and worthless [30]. Individuals with dementia need to gain a sense of belonging [28]. Building meaningful relationship with PWD does have a great impact on maintaining their dignity and personhood. Personhood and dignity can be ensured only within the context of a mutually recognizing, respecting, and trusting relationship. The core of the RCC is maintaining the dignity and personhood of PWD, which is also one of the ethics of nursing [31]. Building reciprocal relationships with PWD and maintaining the dignity and personality of PWD are important components of the RCC. RCC can improve the quality of the dementia care of nursing homes.

The management of BPSD is highly individualized [32]. The complexity of these neuropsychiatric symptoms means that there is no “one size fits all solution,” and approaches tailored to the patient and the caregiver are needed. Due to the specificity of the symptom, the staff need to know the professional management strategies, such as the detailed assessment of the symptoms, treatment approach and evidence for management of BPSD by category [9]. Be positive means that the staff could develop the effective measures by themselves and finally be professional in dementia care through practice research. It also requires caregivers to be positive with PWD and take personalized measures for individual. Training program for dementia care staff usually focused on the care competence and attitudes, that means dementia care staff should be competent to deal with difficult symptoms for PWD independently [17, 33, 34]. Thus, management of the care challenges and positive attitude toward PWD are the essential competence during nursing practice.

The two factors formed in two sub-scales respectively in the SCIDS-C scale are completely in conformity with qualitative-centered care components in high-quality care practice in dementia care and are suitable for the evaluation of nursing staff's dementia care competence. Seventeen items of the SCIDS-C scale are brief and understandable, which is easy to use in nursing staff. Considering the low degree of education state in staff, the responsive options were translated with plain words in semantic equivalence, which can accurately measure the self-competence in nursing staff.

## Conclusion

This study is among the first efforts to evaluate the psychometric characteristic of the SCIDS scale in Chinese frontline dementia care staff. The Chinese version of SCIDS is a culturally sensitive and reliable tool to use in China through our adaptation and testing. The scale is valuable as it provides dementia care staff with a tool for assessing their self-competence during work and evaluating the effects of training program for professional dementia care. Thus, the frontline dementia care staff may exert efforts to increase their care competence to improve PWD’s quality of life and the quality of their own work and life in residential care settings.

Psycholinguistic and psychometric measurements showed that the SCIDS-C scale is applicable to Chinese frontline dementia care staff and determined a homogeneous construct. The Cronbach’s alpha values and intraclass correlation coefficients were high and acceptable. Hence, the SCIDS-C scale is a valid and reliable tool for evaluating the dementia care competence of frontline staff in facilities in the Chinese health care setting.

## Limitation and recommendation

First, the data is comprised of self-reported feedback survey. Self-reporting allows for bias because the staff may inaccurately report outcomes. It is recommended that the researchers should give some instructions to the respondents about the self-report standard. During this research, since the construct of competence can be considered quite stable, one-week interval seems relatively short. Meanwhile, the research did not include participants from rural areas in China. The results might not be generalizable to long-term care setting in rural China. In statistics, Cronbach’s alpha value is affected by the length of the scale so that instruments that have more than 15 items may show high values even if the items reflect different underlying constructs. Moreover, a sample of 174 participants may be quite small for the factor analysis and random results could be possible. So, further research can proceed towards including larger sample size and involving multicenters in both urban and rural areas.

## Data Availability

Data not available due to ethical restrictions. Participants of this study did not agree for their data to be shared publicly, so supporting data is not available. If someone has the question about the data on this study, please contact with the author Xueli Bian. Email: bian.xueli@zs-hospital.sh.cn.
